# The impact of bloodstream infection in patients undergoing appendectomy due to acute appendicitis

**DOI:** 10.1016/j.sipas.2022.100108

**Published:** 2022-07-09

**Authors:** Akina Shinkura, Kenya Yamanaka, Makoto Kurimoto, Hikaru Aoki, Yusuke Hanabata, Kaichiro Harada, Masashi Kayano, Misaki Tashima, Jun Tamura

**Affiliations:** Department of Surgery, Hyogo Prefectural Amagasaki General Medical Center, 2-17-77, Higashinaniwa, Amagasaki, Hyogo, Japan

**Keywords:** Bloodstream infection, Acute appendicitis, Sepsis, Intraabdominal infection

## Abstract

Introduction: Current severity classification of appendicitis does not take into account the impact of bloodstream infection (BSI), despite the importance of BSI in critical care settings. Therefore, we examined whether BSI in emergency appendectomy indicated for acute appendicitis has a clinical impact. Methods: We retrospectively reviewed patients with acute appendicitis who underwent emergency surgery at our department between July 2015 and January 2020. Results: BSI was detected in 14 out of 154 patients (9%). Patients with BSI had a higher rate of major complications, readmission rate, and longer postoperative hospital stay, but there was no difference in the rate of complicated and uncomplicated appendicitis. Multivariate analysis showed age ≧ 58 years (Odds ratio [OR] 8.98 95% Confidence interval [CI] 2.07–39.0) and bilirubin ≧ 2.0 mg/dl (OR 8.79 95% CI 1.09–71.0) were associated with BSI. In 4 out of 8 cases (50%), the bacteria isolated in patients with BSI were not isolated in patients with intraabdominal infection (IAI). Conclusion: BSI in acute appendicitis had a clinical impact on postoperative outcomes, irrespective of complicated and uncomplicated appendicitis. Blood cultures should be considered for older patients, and patients with high bilirubin levels in emergency appendectomy for acute appendicitis.

## 1 Introduction

Emergency appendectomy for acute appendicitis is one of the most common emergency surgery performed worldwide, and has a good prognosis with a morbidity rate of 9.1% and mortality rate of 0.04% [1]. However, the severity of appendicitis ranges widely from mild to severe. Present studies use the classification of complicated/uncomplicated appendicitis [2] and the American Association for the Surgery Trauma (AAST) grading system [3] to assess appendicitis severity. Complicated/uncomplicated appendicitis is the most well-known severity classification of appendicitis. Complicated appendicitis is defined as appendicitis with perforation, abscess formation, and gangrenous appendicitis, whereas uncomplicated appendicitis is defined as simple appendix inflammation including catarrhal and phlegmonous appendicitis [2]. High white blood cell (WBC) count, high C-reactive protein (CRP) level, body temperature ≧38°C, duration of symptoms ≧48 h, and CT findings such as extraluminal free air are known predictors of complicated appendicitis, and are used as scoring systems to distinguish between uncomplicated and complicated appendicitis [4]. AAST grading system is another severity classification of appendicitis, which classifies appendicitis into five categories based on clinical symptoms, image findings, surgical findings, and postoperative pathology [3]. These classifications correlate with postoperative outcomes and are used to assess disease severity [1].

On the other hand, bloodstream infection (BSI) is a vital problem in critical care settings, such as in emergency departments. BSI has been considered a classic marker of the severity of diseases and a contributor to patient mortality [5]. BSI was detected in 13.2% of patients who visited the emergency department with infection, and 7.5% was due to abdominal infection [5]. However, the rate of BSI in acute appendicitis is still unknown. Despite the clinical significance of BSI in daily practice, few studies have investigated its impact on acute appendicitis. Moreover, current classifications of acute appendicitis do not take BSI into consideration. Therefore, we retrospectively examined whether BSI in acute appendicitis indicated for emergency appendectomy has an impact on preoperative clinical characteristics and postoperative outcomes. In addition, we compared blood culture results with intra-abdominal fluid culture results.

## 2 Material and methods

### 2.1 Study design and setting

This retrospective cohort study reviewed the patients aged ≧ 15 years who were diagnosed with acute appendicitis and underwent emergency surgery in Hyogo Prefectural Amagasaki General Medical Center between July 2015 and January 2020. Among 280 patients who underwent surgery with a diagnosis of appendicitis, 154 patients had gone emergency surgery within 24 h after hospital visit. These 154 patients were tested for blood cultures before surgery and were diagnosed with acute appendicitis based on postoperative histopathology. The Ethics Committee of the Hyogo Prefectural Amagasaki General Medical Center approved this study (1–150).

### 2.2 Data collection

The demographic data (age and sex), comorbidities (diabetes), preoperative body temperature, initial laboratory test results (neutrophil percentage, WBC count, platelet count, total bilirubin, creatinine, pH, base excess, and CRP), American Society of Anesthesiologists Physical Status (AS-PS), and the presence of sepsis in each patient were determined. In addition, surgical factors, such as surgical approach, operative time, and blood loss were also collected. The rate of complications and readmissions, length of postoperative hospital stay, and changes in antibiotic regimen were assessed as postoperative outcomes. Postoperative histopathology, blood culture results, and intra-abdominal fluid culture results of the patients were also recorded.

### 2.3 Definitions

*BSI*: Clinicians took blood cultures as their basis. All blood cultures were tested before the initial antimicrobial therapy and before surgery. BSI was defined as when two sets of blood cultures grew the same microorganisms or one set of blood culture had bacteria other than organisms deemed to be considered contaminant, such as coagulase negative *staphylococci, Corynebacterium* species, and *Propionibacterium acnes*.

*Intra-abdominal infection (IAI)*: Surgeons took the intraoperative samples of peri‑appendiceal fluids, including ascites, purulent peritoneal fluid, or intra-abdominal abscess, based on their decision. IAI was defined when microorganisms grew on cultures.

*Severity of appendicitis*: The severity of appendicitis was classified into two categories (complicated and uncomplicated) based on intraoperative macroscopic appearance and postoperative histopathology. Complicated appendicitis was defined as appendicitis with perforation, abscess, or gangrenous appendicitis. Uncomplicated appendicitis was defined as catarrhal appendicitis or phlegmonous appendicitis [2].

*Sepsis:* Patients were identified as septic if they had an acute change (≧ 2 points) in the total Sequential Organ Failure Assessment score consequent to the infection according to the Sepsis-3 campaign [6,7].

*Major complication*: A major postoperative complication was defined as a complication with a Clavien-Dindo classification of Grade ≧ 3 [8].

*Length of hospital stay*: The length of postoperative hospital stay was counted as days from surgery until discharge.

*Change in antibiotics*: Antibiotic escalation was defined as the addition of a new antibiotic or the switching to a broader-spectrum agent, whereas antibiotic streamlining was defined as the narrowing of antibiotics spectrum or the switching to an oral regimen, based on microbiological data obtained [9].

### 2.4 Statistical analyses

Continuous variables were expressed as means and standard deviations and analyzed using student's *t*-test. Categorical variables were expressed as numbers (%) and analyzed using χ2 test. All *p*-values were two-sided, and *p*-values < 0.05 were considered statistically significant. Receiver-operating characteristic curve (ROC) analysis was used to identify optimal cutoff points of the continuous variable with the statistically significant difference. It was defined as the value that indicated the highest sum of sensitivity and specificity on the ROC curve. Univariate and multivariate logistic regression analyses were performed to find the association between preoperative variables and BSI. Length of postoperative hospital stay was estimated using the Kaplan-Meier method and compared using a log-rank test. Statistical analyses were performed using the JMP 8.0 software (SAS Institute, Cary, NC, USA).

## 3 Results

### 3.1 Patient characteristics

BSI was detected in 14 out of 154 patients (9%). Patient characteristics are shown in Table 1. The mean age was 47.3 years and male/female, 86/68. Mean CRP was 8.3 mg/dl, and 27 (18%) patients were diagnosed with sepsis. Surgical approaches were laparoscopic appendectomy, open appendectomy, and others in 134 (87%), 18 (12%), and 2 (1%) respectively, predominantly by laparoscopic appendectomy. Postoperative histopathology was catarrhal, phlegmonous, and gangrenous in 4 (3%), 45 (29%), and 105 (68%), respectively. Complicated appendicitis was seen in 116 (75%) patients. Among 51 patients with obtained intra-abdominal fluid cultures, 9 (6%) patients were negative, and 42 (27%) were positive. Postoperative complications were seen in 27 (18%) patients, and the median hospital stay was 5.5 days. Antibiotics were switched in 11 (7%) patients, including 6 cases of escalation and 5 cases of streamlining.

**Table 1:** Patient characteristics.

	*N* = 154
Age	47.3 ± 20.7
Male, n (%)	86 (56)
Preoperative body temperature, °C	37.3 ± 0.84
White blood cell count, × 10^9^/L	13.2 ± 4.2
Neutrophil,%	84.2 ± 8.7
Platelet count, × 10^9^/L	232 ± 66
T-Bil, mg/dl	1.22 ± 0.70
CRE, mg/dl	0.77 ± 0.31
pH	7.4 ± 0.1
Base excess	0.89 ± 2.26
CRP, mg/dl	8.3 ± 9.5
Diabetes, n (%)	9 (6)
ASA-PS, n (%)	
1E	61 (40)
2E	79 (51)
3E	14 (9)
Sepsis, n (%)	27 (18)
Appendectomy, n (%)	
Laparoscopic	134 (87)
Open	18 (12)
Others	2 (1)
Operative time, min	88.8 ± 40.5
Blood loss, mL	16.2 ± 59.6
Postoperative histopathology, n (%)	
Catarrhal	4 (3)
Phlegmonous	45 (29)
Gangrenous	105 (68)
Complexity, n (%)	
Complicated	116 (75)
Uncomplicated	38 (25)
Blood culture, n (%)	
Negative	140 (91)
Positive	14 (9)
Intra-abdominal fluid culture, n (%)	
Not obtained	103 (67)
Negative	9 (6)
Positive	42 (27)
Postoperative complications, n (%)	27 (18)
Clavian-Dindo Grade≧3, n (%)	5 (3)
Readmissions, n (%)	4 (3)
Postoperative hospital stay, days (95%CI)	5.5 (5 - 6)
Antibiotics switched, n (%)	11 (7)

BSI: bloodstream infection, T-Bil: total bilirubin, CRE: creatinine, CRP: C-reactive protein, ASA-PS: American Society of Anesthesiologists-physical status, CI: confidence intarval.

### 3.2 Comparison of clinical variables between BSI positive and negative patients

The clinical variables and postoperative outcomes of BSI positive and negative patients were compared in Table 2. Age was higher in patients with BSI compared with patients without BSI (*P* = 0.032). Total bilirubin levels, creatinine levels, and the rate of sepsis were higher in patients with BSI compared to patients without BSI (*P* = 0.028, 0.037, and 0.009, respectively). Patients with IAI were significantly higher in patients with BSI (*P* = 0.026). On the other hand, there was no difference in preoperative body temperature, WBC count, Neutrophil percentage, CRP levels, ASA-PS, postoperative pathology, and severity of appendicitis between the two groups. There was no difference in total complications, but the rate of major complications was higher in patients with BSI (*P* = 0.015). Moreover, patients with BSI had a higher readmission rate (*P* = 0.004) and longer length of postoperative hospital stay (*P* < 0.001). The antibiotic regimens were changed in some of the patients with BSI, but none in patients without BSI (*P* < 0.001).

**Table 2:** Differences of clinical variables between BSI positive and negative patients.

	Positive *N* = 14	Negative *N* = 140	*P* values
Age	46.2 ± 20.4	58.6 ± 21.2	0.032*
Male, n (%)	7 (50)	79 (56.4)	0.644
Preoperative body temperature, °C	37.5 ± 1.00	37.4 ± 0.83	0.745
White blood cell count, × 10^9^/L	12.4 ± 6.1	13.2 ± 4.0	0.455
Neutrophil,%	84.0 ± 9.8	84.2 ± 8.6	0.920
Platelet count, × 10^9^/L	247 ± 115	231 ± 60	0.381
T-Bil, mg/dl	1.61 ± 1.20	1.18 ± 0.62	0.028*
CRE, mg/dl	0.93 ± 0.38	0.76 ± 0.30	0.037*
pH	7.4 ± 0.1	7.4 ± 0.0	0.500
Base excess	0.14 ± 2.26	0.97 ± 2.24	0.228
CRP, mg/dl	12.0 ± 7.2	7.9 ± 9.6	0.128
Diabetes, n (%)	0 (0)	9 (6%)	0.328
ASA-PS, n (%)			
1E	5 (36)	56 (40)	
2E	8 (57)	71 (51)	
3E	1 (7)	13 (9)	0.893
Sepsis, n (%)	6 (43)	21 (15)	0.009*
Appendectomy, n (%)			
Laparoscopic	12 (86)	122 (87)	
Open	2 (14)	16 (11)	
Others	0 (0)	2 (2)	0.776
Operative time, min	88.1 ± 34.0	82.2 ± 41.1	0.603
Blood loss, mL	36.4 ± 129	14.2 ± 48.1	0.186
Postoperative histopathology, n (%)			
Catarrhal	0 (0)	4 (3)	
Phlegmonous	2 (14)	43 (31)	
Gangrenous	12 (86)	93 (66)	0.321
Complexity, n (%)			
Complicated	12 (86)	104 (74)	
Uncomplicated	2 (14)	36 (26)	0.344
Intra-abdominal fluid culture, n (%)			
Not obtained	6 (43)	97 (70)	
Negative	0 (0)	9 (6)	
Positive	8 (57)	34 (24)	0.026*
Postoperative complications, n (%)	5 (36)	22 (16)	0.061
Clavian-Dindo Grade≧ 3, n (%)	2 (14)	3 (2)	0.015*
Readmissions, n (%)	2 (14)	2 (1)	0.004*
Postoperative hospital stay, days	14.5	5.5	<0.001*
Antibiotics switched, n (%)	11 (79)	0 (0)	<0.001*

BSI: bloodstream infection, T-Bil: total bilirubin, CRE: creatinine, CRP: C-reactive protein, ASA-PS: American Society of Anesthesiologists-physical status, *: *P* < 0.05.

### 3.3 Univariate and multivariate analysis of preoperative variables to evaluate association with BSI

The univariate and multivariate analyses were performed using the following four variables: age, total bilirubin levels, creatinine levels, and sepsis, which showed a significant association with BSI. The cut-off age was 58 years, cut-off total bilirubin levels was 2.0 mg/dl, and cut-off creatinine levels was 0.66 mg/dl. The results are shown in Table 3. In the multivariate analysis, age ≧ 58 years (Odds ratio [OR] 8.98 95% confidence interval [CI] 2.07–39.0) and total bilirubin levels ≧ 2.0 mg/dl (OR 8.79 95% CI 1.09–71.0) were associated with BSI, whereas Creatinine level and sepsis were not associated with BSI.

**Table 3:** Associated preoperative variables of BSI in acute appendicitis.

	Univariate analysis			Multivariate analysis		
	OR	95% CI	*P* value	OR	95% CI	*P* value
Age ≥ 58	6.04	1.79–20.4	0.004*	8.98	2.07–39.0	0.003*
T-Bil≥ 2.0	4.63	1.37–15.6	0.014*	8.79	1.09–71.0	0.041*
Cre ≥ 0.66	7.00	0.89–55.1	0.065	9.20	0.95–88.6	0.055
Sepsis, yes	4.25	1.34–13.5	0.014*	0.95	0.16–5.56	0.953

BSI: bloodstream infection, OR; odds ratio, CI; confidence interval, T-Bil: total bilirubin, CRE: creatinine, *; *P* < 0.05.

### 3.4 Bacterial species isolated in patients with BSI and IAI

A comparison between bacterial species of blood cultures and intra-abdominal fluid cultures isolated in patients with BSI is shown in Table 4. In 14 cases of BSI, 19 bacteria (10 species) were isolated, including 9 *Bacteroides* species (4 *Bacteroides fragilis [B. fragilis]*, 2 *Bacteroides thetaiota [B. thetaiota]*, and 3 others), 2 *Escherichia coli (E. coli)*, 2 *Eubacterium lentum (E. lentum)*, 2 anaerobic Gram-positive rod (GPR), 1 anaerobic Gram-negative rod (GNR), 1 *Enterobacter aerogenes (E. aerogenes)*, 1 *Gemella* species, and 1 *Bacillus* species. Among 14 patients with BSI, intra-abdominal fluid cultures were taken in 8 patients. In 8 cases of IAI, 25 bacteria (10 species) were isolated, including 7 *Bacteroides* species (3 *B fragilis*, 2 *B thetaiota*, and 2 others), 4 *E. coli*, 3 *Streptococcus* species, and others. In 4 out of 8 cases (50%), the bacteria isolated in patients with BSI were not isolated in patients with IAI.

**Table 4:** Comparison of pathogens isolated in IAI and BSI.

BSI	IAI	Age, year	T-Bil, mg/dl
E. coli, Anaerobic GPR, E. lentum	Bacteroides spp., E. coli, Anaerobic GNR, Peptostreptococcus spp.	37	0.9
B. fragilis	B. fragilis, Peptostreptococcus spp., Streptococcus spp.	16	0.4
Anaerobic GNR	B. thetaiota, E. coli, Bacillus spp.	71	2.2
Bacillus spp.	B. thetaiota, E. coli, P. aeruginosa, Bacillus spp.	60	1.2
Bacteroides spp.	Bacteroides spp., K. pneumoniae, M. morganii, E. faecalis, E. raffinosus	80	1
B. fragilis, Bacteroides spp., E. lentum	B. fragilis, α-streptococcus	78	0.5
B. fragilis	B. fragilis, K. pneumoniae, Streptococcus spp.	89	0.9
E. coli	E. coli	64	0.5
B. thetaiota	(Not obtained)	76	1.6
Anaerobic GPR	(Not obtained)	64	2
Bacteroides spp.	(Not obtained)	58	4.7
B. fragilis, Gemella spp.	(Not obtained)	28	2.1
B. theraiota	(Not obtained)	60	1.2
E. aerogenes	(Not obtained)	40	3.3

IAP: intra-abdominal infection, BSI: bloodstream infection, E. coli: Escherichia coli, GNR: Gram negative rod, GPR: Gram positive rod, E. lentum: Eubacterium lentum, B. fragilis: Bacteroides fragilis, B. thetaiota: Bacteroides thetaiota, P. aeruginosa: Pseudomonas aeruginosa, K. pneumoniae: Klebsiella pneumoniae, M. morganii: Morganella morganii, E. faecalis: Enterococcus faecalis, E. raffinosus: Enterococcus raffinosus, E. avium: Enterococcus avium, E. aerogenes: Enterobacter aerogenes.

## 4 Discussion

The most common severity classification of acute appendicitis is uncomplicated and complicated appendicitis. Interestingly however, this study showed no significant association between BSI and complicated/uncomplicated appendicitis. On the other hand, we found the following clinical impact of BSI in acute appendicitis. The rate of major complications was higher in patients with BSI (*P* = 0.015), patients with BSI had a higher readmission rate (*P* = 0.004), and longer length of postoperative hospital stay (*P* < 0.001). Therefore, BSI in acute appendicitis indicated for acute appendectomy has a clinical impact on postoperative outcomes, irrespective of complicated and uncomplicated appendicitis.

In this study, BSI was detected in 9% of patients with acute appendicitis who underwent emergency surgery; not all patients with appendicitis require preoperative blood culture tests. The multivariate analysis showed that age ≧ 58 years and total bilirubin levels ≧ 2 mg/dl were associated with BSI. Although it is not limited to acute appendicitis, age ≧ 60 years, female gender, heart rate ≧ 90 bpm, high neutrophil percentage are predictive factors of BSI in general medical inpatients [10]. A study targeted for acute cholecystitis shows age ≧ 72 years and grade 3 acute cholecystitis are associated with BSI [11]. Total bilirubin level is also reported as a biomarker for BSI [12]. Although we could not find that sepsis was statistically significant different between patients with and without BSI in the multivariate analysis, it is reported that septic shock is highly associated with BSI [13]. Therefore, taking blood cultures should be considered for older patients and high serum Bilirubin levels in case of emergency appendectomy.

IAI was significantly higher in patients with BSI (*P* = 0.026) in this study, suggesting that BSI might be linked with IAI. Most of the bacterial species isolated in patients with IAI were *E. coli* and *Bacteroides* species, and their frequencies are compatible to previous reports [14,15]. However, the bacterial profiles of patients with BSI and IAI were different. The frequency of *E. coli* was 16.0% in patients with IAI and 10.5% in patients with BSI. The frequency of *Bacteroides* species was 28.0% in patients with IAI and 47.4% in patients with BSI. In addition, in 4 out of 8 cases (50%), the bacteria isolated in patients with BSI were not isolated in patients with IAI. Moreover, the antibiotic regimens were changed in 11 patients with BSI, but none in patients without BSI. Blood culture does have a clinical significance for patients with BSI who undergo emergency appendectomy.

This study has several limitations. Because this is a single-site retrospective cohort study with a small sample size, it includes possible biases and lacks a control group. Moreover, whether to obtain a blood culture and intra-abdominal fluid culture was only based on the clinicians’ decisions, and there are cases without culture samples. There were also no criteria for the emergency surgery of acute appendicitis, and it was only based on the clinicians’ decision to undergo surgery. Finally, this study did not examine the indications for collecting blood culture in patients with acute appendicitis, and BSI cannot be diagnosed preoperatively. We believe that solving these problems is needed to establish the clinical significance of blood culture in the future.

## 5 Conclusion

In conclusion, BSI in acute appendicitis had a clinical impact on postoperative outcomes, irrespective of complicated and uncomplicated appendicitis. Although IAI was significantly higher in patients with BSI, the bacteria isolated in patients with BSI were not isolated in patients with IAI with a high rate. Therefore, blood cultures should be considered for older patients and high serum total bilirubin levels in emergency appendectomy for acute appendicitis.

## Declaration of Competing Interest

The authors declare that they have no known competing financial interests or personal relationships that could have appeared to influence the work reported in this paper.

## Funding

This research did not receive any specific grant from funding agencies in the public, commercial, or not-for-profit sectors.
